# Supplementary material for: Prunus spinosa Extract Loaded in Biomimetic Nanoparticles Evokes In Vitro Anti-Inflammatory and Wound Healing Activities
Source: Nanomaterials (Basel). 2020 Dec 25;11(1):36. doi: 10.3390/nano11010036 (PMC7824340; doi:10.3390/nano11010036)
Supplement: Supplementary file 1 [file nanomaterials-11-00036-s001.pdf]

nanomaterials

MDPI

Supplementary material of

Prunus spinosa extract loaded in biomimetic nanoparticles evokes *in vitro* anti-inflammatory and wound healing activities

Mattia Tiboni[1a*], Sofia Copparia[1a], Luca Casettari[1], Michele Guescini[1], Mariastella Colomba[1], Daniele Fraternale[1], Andrea Gorassini[2], Giancarlo Verardo[3], Seeram Ramakrishna[4], Loretta Guidi[1], Barbara Di Giacomo[1], Michele Mari[1], Roberto Molinaro[1, 5*] and Maria Cristina Albertini[1]

[1] Department of Biomolecular Sciences, University of Urbino Carlo Bo, 61029 Urbino (PU), Italy; mattia.tiboni@uniurb.it (M.T.); s.coppari3@campus.uniurb.it (S.C.); luca.casettari@uniurb.it (L.C.); mariastella.colomba@uniurb.it (M.G.); mariastella.colomba@uniurb.it (M.C.); daniele.fraternale@uniurb.it (D.F.); loretta.guidi@uniurb.it (L.G.); barbara.digiacomo@uniurb.it (B.D.G.); michele.mari@uniurb.it (M.M.)

[2] Department of Humanities and Cultural Heritage, University of Udine, 33100 Udine, Italy; andrea.gorassini@uniud.it

[3] Department of Agricultural, Food, Environmental and Animal Sciences, University of Udine, 33100 Udine, Italy; giancarlo.verardo@uniud.it

[4] Center for Nanofibers and Nanotechnology, National University of Singapore, Singapore 119077, Singapore; mpesr@nus.edu.sg

[5] IRCCS Ospedale San Raffaele srl, 20132 Milan, Italy

* Correspondence: molinaro.roberto@hsr.it (R.M.); maria.albertini@uniurb.it (M.C.A.); Tel.: +39-0226434987 (R.M.); +39-0722305260 (M.C.A.)

† Equally contributed authors.

1. Methods

1.1 Analysis of P. spinosa extract, PS-DOPC-Leukosome, and PS-DOPG-Leukosome by HPLC-DAD-ESI-MS^n.

P. spinosa ethanolic extract, PS-DOPC-Leukosome, and PS-DOPG-Leukosome were purified by solid-phase extraction (SPE). To this purpose each sample (5.0 mg) was shaken with H_2O containing 0.4% formic acid (2 mL) in a vortex mixer for about 15 min and loaded on a ISOLUTE C18 column pre-conditioned by sequentially passing 5 mL of MeOH with 0.4% formic acid and 5 mL of H_2O with 0.4% formic acid. After loading the sample, the column was washed with 5 mL of H_2O containing 0.4% formic acid, and the phenolic fraction was eluted with 10 mL of MeOH-H_2O (7:3, v/v) with 0.4% formic acid. The solvents were removed under vacuum at 30 °C, and the residue was dissolved again in 1 mL of H_2O/MeOH (9:1, v/v) with 0.4% formic acid for the HPLC-DAD-ESI-MS^n analysis. Chromatographic analysis was performed with a Dionex Ultimate 3000 UHPLC (Thermo Scientific, San Jose, CA, USA) equipped with a thermostated autosampler and a column oven. The chromatographic separation was obtained with a column Synergi Hydro, 4 μm, 250 × 2.0 mm (Phenomenex, Italy), thermostated at 30 °C. Elution was carried out at a flow rate of 0.3 mL/min, using as a mobile phase a mixture of 0.2% formic acid in methanol (A) and 0.2% formic acid in water (B) with the following gradient: 0-6 min 10% A, 20 min 40% A, 40 min 40% A, 46 min 100% A, 56 min 100% A, 58 min 10% A, and 58-65 min 10% A. The injection volume was 20 μL. The UHPLC system was coupled with a diode array detector and an electrospray ionization mass detector (HPLC-DAD-ESI-MS^n) in parallel by splitting the mobile phase 1:1.

ESI mass spectra were obtained with a Finnigan LXQ linear trap mass spectrometer (Thermo Scientific, San Jose, CA, USA). The typical ESI source conditions were transfer line capillary at 275 °C; ion spray voltage at 3.30 kV; sheath, auxiliary and sweep gas (N_2) flow rates at 50, 10 and 0 arbitrary units, respectively. Helium was used as the collision damping gas in the ion trap set at a pressure of 0.13 Pa. The acquisition was carried out in full scan (*m*/*z* 50–1500) and in full scan MS^2 (*m*/*z* 50–800) selecting both positive and negative precursor ions.

The characterization of phenolic compounds was carried out by comparison of their fragmentation pattern with authentic standards and/or with data available in the literature. Table S1 shows the chromatographic retention times, MS^n fragmentation ions, and UV-Vis spectra of all tentatively identified compounds.

1.2 Calibration curves and quantification of encapsulated P. spinosa extract

The quantitative analysis was carried out using an Ultimate 3000 RS Diode Array detector (Thermo Scientific, San Jose, CA, USA) controlled by Chromeleon software (version 6.80). Spectral data from all peaks were accumulated in the range of 200–600 nm. The quantification of the phenolic compounds was carried out by external calibration from the areas of the chromatographic peaks obtained by UV detection at the following wavelengths: 258 nm for flavones and flavonols, 280 nm for hydroxybenzoic acid derivatives, 328 nm for hydroxycinnamic acid derivatives, and 520 nm for anthocyanins. A stock solution of chlorogenic acid, gallic acid, quercetin-3-O-galactoside, quercetin-3-O-xyloside, quercetin-3-O-arabinoside, quercetin-3-O-rhamnoside, cyanidin chloride in H_2O/MeOH (9:1, v/v) with 0.4% formic acid was serially diluted with the same solvent to prepare calibration curves ranging from 12-3000 ng/mL. The R^2 coefficients for the calibration curves were > 0.99. When standards were unavailable, the quantification of the analytes were carried out using the calibration curve of available standard presenting similar chemical structures: quercetin 3-O-rhamnoside, quercetin 3-O-hexoside-O-pentoside, and rutin were quantified with the calibration curve of quercetin 3-O-rhamnoside (wavelength max 258 nm); quercetin arabinoside, apigenin pentoside, apigenin pentoside isomer, and quercetin pentoside as quercetin arabinoside (wavelength max 258 nm); quercetin galactoside and quercetin hexoside as quercetin galactoside (wavelength max 258 nm); 4-(vanilloyloxy)-2,6,6-trimethylcyclohexene-1-carboxylic acid and ellagic acid as gallic acid (wavelength max 280 nm); 3-O-caffeoylquinic acid, 3-O-p-cumaroylquinic acid, chlorogenic acid dehydrodimer, 3-O-feruloylquinic acid, 4-O-caffeoylquinic acid, and chlorogenic acid dehydrodimer

as chlorogenic acid (wavelength max 328 nm); and cyanidin 3-O-glucoside, cyanidin 3-O-rutinoside, peonidin 3-O-glucoside, and peonidin 3-O-rutinoside as cyanidin chloride (wavelength max 520 nm). The samples were analysed in triplicate.

2. Results

2.1 Analysis of P. spinosa extract, PS-DOPC-Leukosome and PS-DOPG-Leukosome by HPLC-DAD-ESI-MS^n.

Among the 24 compounds listed in Table S1, peaks 1-12, 15, 17-24 were identified by comparing their retention time, UV data, and MS^n fragmentation pattern with those of authentic standards and/or with data available in the literature. Peak 16 showed a $[M-H]^-$ ion at *m/z* 333 and produced fragments (MS^2) at *m/z* 165, 289, and 301 due to the loss of vanillic acid (168 Da), CO_2 (44 Da), and MeOH (32 Da), respectively. On the base of the fragmentation pattern and literature data on picrocrocinic acid [1–3], peak 16 was tentatively identified as 4-(vanilloyloxy)-2,6,6-trimethylcyclohexene-1-carboxylic acid. The compounds corresponding to peaks 13 and 14 were unidentified.

Table S1. Characterization of the main phenolic compounds of the extracts of purified samples of *P. spinosa* ethanolic extract by HPLC–DAD/ESI–MS^n in positive or negative mode.

Peak No	t_R (min)	λ_{max} (nm)	M^+ or $[M+Na]^+$ (m/z)	$[M-H]^-$ or $[M+HCOO]^-$ (m/z)	HPLC-ESI/MS^n *m/z* (% base peak)	Tentative assignment	Ref.
1	14.6	300sh, 326		353	MS^2[353]: 191 (100), 179 (44), 135 (8)	3-O-Caffeoylquinic acid	c
2	17.6	312		337	MS^2[337]: 163 (100), 191 (9), 173 (6)	3-O-p-Cumaroylquinic acid	[4,5]
3	19.0	295, 320		705	MS^2[705]: 513 (100); MS^3 [705 → 513]: 339 (100); MS^4 [705 → 513 → 339]: 295 (100)	Chlorogenic acid dehydrodimer	[6]
4	19.2	300, 326		367	MS^2[367]: 193 (100), 134 (5), 173 (3), 191 (2)	3-O-Feruloylquinic acid	[4]
5	19.6	300sh,328		353	MS^2[353]: 173 (100), 179 (54), 191 (28), 135 (65)	4-O-Caffeoylquinic acid	[4]
6	20.1	356		447[a]	MS^2[447]: 401 (100); MS^3 [447 → 401]: 269 (100)	Apigenin pentoside	[7]
7	20.4	287, 320		705	MS^2[705]: 513 (100); MS^3 [705 → 513]: 339 (100); MS^4 [705 → 513 → 339]: 295 (100)	Chlorogenic acid dehydrodimer	[6]

8	20.6	520	449		MS^2[449]: 287 (100)	Cyanidin 3-O-glucoside or galactoside	[5]
9	20.9	356		447[a]	MS^2[447]: 401 (100); MS^3 [447 $\rightarrow$ 401]: 269 (100)	Apigenin pentoside isomer	[7]
10	21.4	282, 520	595		MS^2[595]: 287 (100), 449 (20)	Cyanidin 3-O-rutinoside	[8]
11	22.2	271, 302sh, 518	463		MS^2[463]: 301 (100)	Peonidin 3-O-glucoside	[9,10]
12	22.9	282, 522	609		MS^2[609]: 301 (100), 463 (30)	Peonidin 3-O-rutinoside	[9,11]
13	24.9	270, 296	665[b]		MS^2[665]: 357 (100), 633 (31), 647 (3); MS^3 [665 $\rightarrow$ 357]: 173 (100), 191 (15), 189 (12)	Unknown	
14	25.2	270, 360	519[b]		MS^2[519]: 357 (100), 487 (7), 501 (3); MS^3 [519 $\rightarrow$ 357]: 173 (100), 191 (15), 189 (12)	Unknown	
15	27.1	250, 338		609	MS^2[609]: 301 (100), 257 (20); MS^3 [609 $\rightarrow$ 301]: 257 (100), 283(5), 229(1)	Ellagic acid derivative	[11,12]
16	27.8	268, 298		333	MS^2[333]: 165 (100), 301 (30), 289 (10)	4-(vanilloyloxy)-2,6,6-trimethylcyclohexene-1-carboxylic acid	[1–3]
17	27.9	342		463	MS^2[463]: 301 (100)	Quercetin hexoside	[8]
18	31.4	354		595	MS^2[595]: 300 (100), 415 (40), 301 (40), 271 (18), 505 (30), 433 (12)	Quercetin 3-O-hexoside-O-pentoside	[13]
19	31.7	258, 356		609	MS^2[609]: 301 (100), 343 (10)	Rutin	[14]
20	32.4	258, 356		463	MS^2[463]: 301 (100)	Quercetin galactoside	c
21	34.3	258, 354		433	MS^2[433]: 301 (100)	Quercetin xyloside	c
22	34.9	258, 356		433	MS^2[433]: 301 (100), 300 (80)	Quercetin arabinoside	c
23	36.8	258, 356		433	MS^2[433]: 301 (100)	Quercetin pentoside	[15]
24	39.0	260, 352		447	MS^2[447]: 301 (100), 300 (20), 285 (10)	Quercetin 3-O-rhamnoside	c

[a]Formate adduct. [b]Sodium adduct. [c]Confirmed with standard

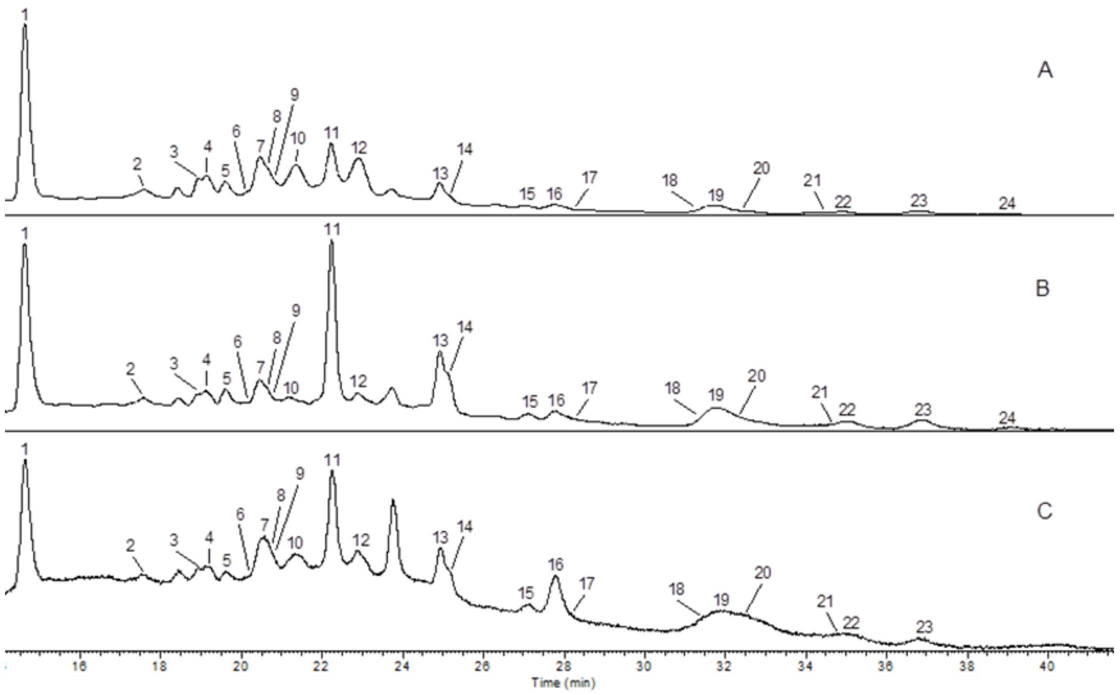

Figure S1. Chromatograms of purified samples of *P. spinosa* ethanolic extract (A), PS-DOPC-Leukosome (B) and PS-DOPG-Leukosome (C) detected at 280 nm. Peak numbers correspond to those reported in Tables S1.

References

1. Wang, L.; Liu, S.; Zhang, X.; Xing, J.; Liu, Z.; Song, F. A strategy for identification and structural characterization of compounds from Gardenia jasminoides by integrating macroporous resin column chromatography and liquid chromatography-tandem mass spectrometry combined with ion-mobility spectrometry. *J. Chromatogr. A* **2016**, *1452*, 47–57, doi:10.1016/j.chroma.2016.05.026.
2. Zou, L.; Li, X.; Shi, Q.; Feng, F. An effective integrated method for comprehensive identification of eighty-five compounds in Zhi-Zi-Da-Huang decoction by HPLC-DAD-ESI-MS (TOF) and HPLC-DAD-ESI-MS/MS (QqQ) without the help of reference standards. *Anal. Methods* **2014**, *6*, 4312–4327, doi:10.1039/c4ay00219a.
3. Liu, Y.; Yang, G.; Feng, F. Integrated chemical profiling of Zhi-Zi-Hou-Po decoction by liquid chromatography-diode array detector-time of flight mass analyzer and liquid chromatography-triple stage quadrupole mass analyzer combined with chemometrics. *Anal. Methods* **2016**, *8*, 4689–4710, doi:10.1039/c6ay01233g.
4. Clifford, M.N.; Johnston, K.L.; Knight, S.; Kuhnert, N. Hierarchical scheme for LC-MSn identification of chlorogenic acids. *J. Agric. Food Chem.* **2003**, *51*, 2900–2911, doi:10.1021/jf026187q.
5. Zhang, J.Y.; Zhang, Q.; Li, N.; Wang, Z.J.; Lu, J.Q.; Qiao, Y.J. Diagnostic fragment-ion-based and extension strategy coupled to DFIs intensity analysis for identification of chlorogenic acids isomers in Flos Lonicerae Japonicae by HPLC-ESI-MSn. *Talanta* **2013**, *104*, 1–9, doi:10.1016/j.talanta.2012.11.012.
6. Castillo-Fraire, C.M.; Poupard, P.; Guilois-Dubois, S.; Salas, E.; Guyot, S. Preparative fractionation of 5′-O-caffeoylquinic acid oxidation products using centrifugal partition chromatography and their investigation by mass spectrometry. *J. Chromatogr. A* **2019**, *1592*, 19–30, doi:10.1016/j.chroma.2019.01.071.
7. Gulsoy-Toplan, G.; Goger, F.; Yildiz-Pekoz, A.; Gibbons, S.; Sariyar, G.; Mat, A. Chemical constituents of the different parts of colchicum micranthum and c. Chalcedonicum and their cytotoxic activities. *Nat.*

Prod. Commun. **2018**, *13*, 535–538, doi:10.1177/1934578x1801300506.

8. Lin, L.Z.; Harnly, J.M. A screening method for the identification of glycosylated flavonoids and other phenolic compounds using a standard analytical approach for all plant materials. *J. Agric. Food Chem.* **2007**, *55*, 1084–1096, doi:10.1021/jf062431s.
9. Wu, X.; Prior, R.L. Systematic identification and characterization of anthocyanins by HPLC-ESI-MS/MS in common foods in the United States: Fruits and berries. *J. Agric. Food Chem.* **2005**, *53*, 2589–2599, doi:10.1021/jf048068b.
10. Schütz, K.; Persike, M.; Carle, R.; Schieber, A. Characterization and quantification of anthocyanins in selected artichoke (Cynara scolymus L.) cultivars by HPLC-DAD-ESI-MS n. *Anal. Bioanal. Chem.* **2006**, *384*, 1511–1517, doi:10.1007/s00216-006-0316-6.
11. Ruiz, A.; Hermosín-Gutiérrez, I.; Vergara, C.; von Baer, D.; Zapata, M.; Hitschfeld, A.; Obando, L.; Mardones, C. Anthocyanin profiles in south Patagonian wild berries by HPLC-DAD-ESI-MS/MS. *Food Res. Int.* **2013**, *51*, 706–713, doi:10.1016/j.foodres.2013.01.043.
12. Topalović, A.; Knežević, M.; Gačnik, S.; Mikulic-Petkovsek, M. Detailed chemical composition of juice from autochthonous pomegranate genotypes (Punica granatum L.) grown in different locations in Montenegro. *Food Chem.* **2020**, *330*, 127261, doi:10.1016/j.foodchem.2020.127261.
13. Masike, K.; Khoza, B.S.; Steenkamp, P.A.; Smit, E.; Dubery, I.A.; Madala, N.E. A metabolomics-guided exploration of the phytochemical constituents of vernonia fastigiata with the aid of pressurized hot water extraction and liquid chromatography-mass spectrometry. *Molecules* **2017**, *22*, doi:10.3390/molecules22081200.
14. Calani, L.; Beghè, D.; Mena, P.; Del Rio, D.; Bruni, R.; Fabbri, A.; Dall'Asta, C.; Galaverna, G. Ultra-HPLC-MSn (poly)phenolic profiling and chemometric analysis of juices from ancient Punica granatum L. cultivars: A nontargeted approach. *J. Agric. Food Chem.* **2013**, *61*, 5600–5609, doi:10.1021/jf400387c.
15. Zhuang, B.; Bi, Z.M.; Wang, Z.Y.; Duan, L.; Lai, C.J.S.; Liu, E.H. Chemical profiling and quantitation of bioactive compounds in Platycladi Cacumen by UPLC-Q-TOF-MS/MS and UPLC-DAD. *J. Pharm. Biomed. Anal.* **2018**, *154*, 207–215, doi:10.1016/j.jpba.2018.03.005.

Publisher's Note: MDPI stays neutral with regard to jurisdictional claims in published maps and institutional affiliations.

© 2020 by the authors. Submitted for possible open access publication under the terms and conditions of the Creative Commons Attribution (CC BY) license (http://creativecommons.org/licenses/by/4.0/).
